# Biomarkers of Selenium Status

**DOI:** 10.3390/nu7042209

**Published:** 2015-03-31

**Authors:** Gerald F. Combs

**Affiliations:** Grand Forks Human Nutrition Research Center, USDA-ARS, 2420 2nd Ave N Grand Forks, ND 58202, USA; E-Mail: gerald.combs@ars.usda.gov; Tel.: +1-(701)-795-8456

**Keywords:** selenium, biomarker, status, antitumorigenesis, toxicity, selenoprotein, selenoprotein P, glutathione peroxidase, selenosugar, methylselenol

## Abstract

The essential trace element, selenium (Se), has multiple biological activities, which depend on the level of Se intake. Relatively low Se intakes determine the expression of selenoenzymes in which it serves as an essential constituent. Higher intakes have been shown to have anti-tumorigenic potential; and very high Se intakes can produce adverse effects. This hierarchy of biological activities calls for biomarkers informative at different levels of Se exposure. Some Se-biomarkers, such as the selenoproteins and particularly GPX3 and SEPP1, provide information about function directly and are of value in identifying nutritional Se deficiency and tracking responses of deficient individuals to Se-treatment. They are useful under conditions of Se intake within the range of regulated selenoprotein expression, e.g., for humans <55 μg/day and for animals <20 μg/kg diet. Other Se-biomarkers provide information indirectly through inferences based on Se levels of foods, tissues, urine or feces. They can indicate the likelihood of deficiency or adverse effects, but they do not provide direct evidence of either condition. Their value is in providing information about Se status over a wide range of Se intake, particularly from food forms. There is need for additional Se biomarkers particularly for assessing Se status in non-deficient individuals for whom the prospects of cancer risk reduction and adverse effects risk are the primary health considerations. This would include determining whether supranutritional intakes of Se may be required for maximal selenoprotein expression in immune surveillance cells. It would also include developing methods to determine low molecular weight Se-metabolites, *i.e.*, selenoamino acids and methylated Se-metabolites, which to date have not been detectable in biological specimens. Recent analytical advances using tandem liquid chromatography-mass spectrometry suggest prospects for detecting these metabolites.

## 1. Introduction

Selenium (Se) has multiple biological activities. The first of these to be recognized was its toxic potential when, in the 1930s, Se was identified as a principal in a plant-induced neuropathy of grazing horses and cattle in the upper Great Plains of the United States [1]. Next to be recognized, in the 1950s, was its nutritional role when experiments with laboratory and livestock species showed that Se prevented pathologies in vitamin E-deficient animals [2,3,4]. The status of nutritional essentiality was extended to humans in the 1980s when very low blood Se levels were associated with an endemic heart disease in parts of China [5] and Se supplementation was found to be preventive [6]. Starting in the 1960s, Se was shown to have anti-tumorigenic capabilities in a variety of animal models of both primary (see review [7]) and secondary tumors [8,9]. Those studies ultimately led to several human clinical trials that have shown cancer risk reduction for subjects of low to moderate Se status [10,11,12,13,14,15,16], but not for subjects of relatively high Se status, e.g., with plasma Se concentrations >120 ng/mL [16,17,18].

In animal models these various roles of Se show different dose-response relationships. Biochemical and physiological lesions of Se deficiency are prevented by relatively low dietary levels, e.g., 0.1 mg/kg [19]. Reductions in experimental tumorigenesis are seen when Se is fed at 10–20 times the nutritional level, e.g., at least 1.5 mg/kg [20,21]. Adverse effects are not seen unless still greater dietary levels are used, e.g., >5 mg/kg [22].

Understanding the health implications of Se in humans has been far more difficult, as Se intakes nor tissue levels of free-living people can seldom be ascertained with the levels of confidence typical of controlled animal experiments. For this reason, Se status in humans and most individual animals must be inferred from measures related to the intake and disposition of Se.

## 2. Selenium Status

**Figure 1:**
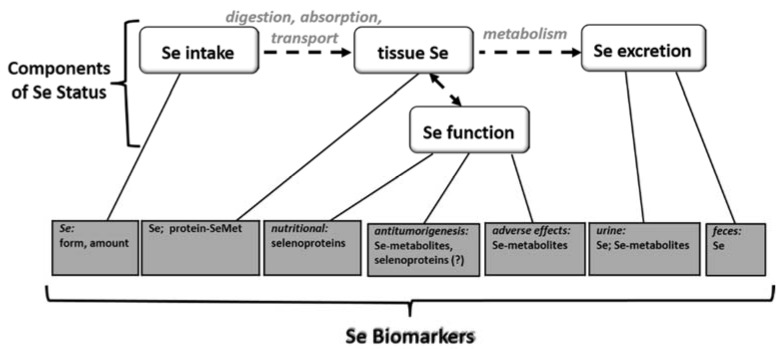
Selenium (Se) biomarkers and their relationships to the components of Se status.

Status is a term of art in the field of nutrition. It refers to the amount of biologically active or potentially active nutrient in the body. Nutritional status is a product of a nutrient’s intake, retention and metabolism. It includes the nutrient pool that is metabolically functional and, thus, the most nutritionally relevant, as well at that pool that can be readily mobilized to functional forms. Accordingly, Se status has four components: Se intake, tissue Se, Se excretion and Se function (Figure 1). Selenium status is assessed for several purposes: to determine the risk of nutritional Se deficiency; to estimate the potential for reducing cancer risk; to monitor the risk of adverse effects associated with excess Se. Such assessments are made in research and clinical care; they may also inform public health program development/evaluation. These purposes can rely on different sets or interpretations of biomarkers.

## 3. Selenium Biomarkers

### 3.1. Biomarkers of Selenium Intake

The Se contents of foods vary according to the soluble Se contents of soils and the capacities of feed plants to take up that Se. Thus, foods of all types show geographic patterns of variation in Se content reflecting, in general, local soil Se conditions at the points of origin. Accordingly, the average daily Se intakes of adults vary among different regions, the lowest being in areas of endemic Se-deficiency diseases in China [23,24]. In most human diets, the main sources of Se are cereals, meats and fish. An analysis of American diets [25] revealed that five foods (beef, white bread, pork, chicken, eggs) contributed half of the total Se in the “typical” diet, and that 80% of the total dietary Se was provided by a core of only 22 foods. The dominant form of Se in foods is thought to be selenomethionine (SeMet). While SeMet can be synthesized only by plants, both plants and animals can incorporate this unusual amino acid nonspecifically into proteins as a mimic for the sulfur-amino acid methionine (Met). Animals and some microorganisms can synthesize SeCys, which they incorporate specifically into a relatively small number of selenoproteins. Therefore, animal products typically contain both SeMet in most proteins and SeCys in selenoproteins.

In general, food Se is well utilized. For the Se-containing amino acids, this involves digestion of their respective proteins, and absorption of the digestive products apparently by active transport processes. For inorganic forms of Se, which do not require digestion, this involves absorption by simple (selenite) or carrier-mediated (selenate) diffusion [26,27], such the fractional absorption of selenate is somewhat better than that of selenite [28]. Wastney *et al.* [29] found that the enteric absorption of single doses (200 μg) by healthy humans were 98% for SeMet-Se and 84% for selenite-Se. Similar results were obtained by Thomson and Robinson [30].

Selenium intakes of individuals or populations can be assessed as the sum of the products of the Se contents of particular foods and the amounts of those foods consumed. In practice, this is done using standard food composition tables and food frequency questionnaires, which may did not yield accurate estimates of actual Se intake because of the geographic variation in food Se contents not captured in food tables. Therefore, determination of food Se by actual analysis of representative samples is necessary to produce Se intake valid estimates. Speciation of Se has been accomplished only for a few foods, e.g., lentil [31]. However, such information would not appear to add value in understanding Se status, as estimates of total Se intake are sufficient to determine whether individuals and populations meet the recommended Se intakes.

**Figure 2:**
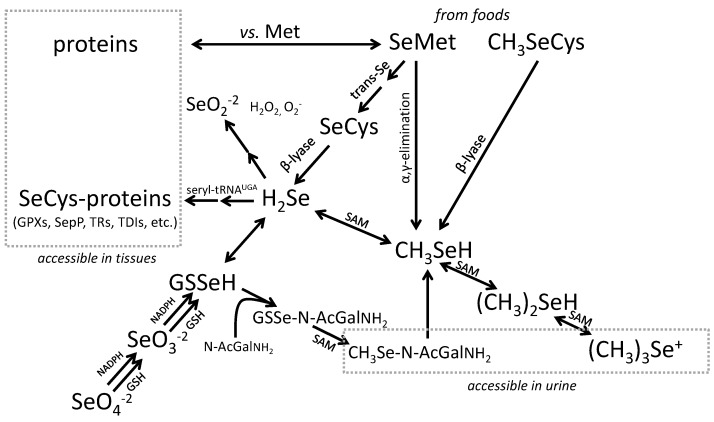
Metabolism of Se. Note that inorganic Se can be incorporated into selenocystaine (SeCys)-containing selenoproteins by way of the obligate intermediate selenide (H_2_Se); while Se from selenomethionine (SeMet) can also be incorporated into the SeCys-containing proteins by way of catabolism to H_2_Se, and into other proteins directly by competing with methionine (Met) in protein synthesis. Other chemical symbols: SeO_2_^−2^ = selenium dioxide; SeO_3_^−2^ = selenite; SeO_4_^−2^ = selenate; CH_3_SeH = methyselenol; (CH_3_)_2_SeH = dimethylselenide’ (CH_3_)_3_Se^+^ = trimethyselenonium ion.

It would be useful to be able to impute Se intake from measured Se biomarkers of Se status if that approach might be less prone to errors of the type inherent in standard food tables. This approach is most straightforward in subjects of deficient to low Se status whose plasma Se concentrations respond to supplemental Se in proportion to the magnitude of supplementation [32]. For non-deficient subjects, however, the relationship of Se intake and plasma Se level depends on the form of Se consumed. Inorganic Se typically produces only minimal increases (<20%) in subjects with plasma Se concentrations greater than *ca.* 70 ng/mL [33,34,35], while SeMet or SeMet-containing foods produce increases in plasma Se in over a wide range including subjects of relatively high Se status [33,34,35,36,37,38,39]. The former effect reflects the fact that the central organ of Se metabolism, the liver, can convert inorganic Se to forms incorporated only into selenoproteins (Figure 2), including two in the plasma, the extracellular glutathione peroxidase (GPX3) and the Se-transporter selenoprotein P (SEPP1) that show plateaued expression at Se intakes recommended as being nutritionally adequate. The latter, continuing effect is due to the unregulated, non-specific incorporation of SeMet into albumin and other proteins over a virtually unlimited range of intakes [39,40,41,42,43,44]. For Se-nondeficient individuals consuming Se mostly as SeMet, Se intake can be predicted from the Se concentration of plasma (see Section 3.2).

### 3.2. Biomarkers of Selenium Retention

Most absorbed Se is taken up by liver to re-enter the circulation as a component of SEPP1, the primary transporter to peripheral tissues [45]. A significant amount (15%–20%) is released in the urine over several days. Under normal conditions, there appears to be only a small enterohepatic circulation of absorbed Se; therefore, fecal Se is comprised mostly of the unabsorbed portion of ingested Se. In animals fed nutritionally adequate levels of selenite, fecal Se comprises about a fifth of Se excretion [46]; fecal Se increases at higher Se intakes.

Therefore, at nutritional intakes, Se retention in the body can be determined by the difference between that amount of Se ingested and the sum of urinary and fecal Se. For humans this requires total urine and stool collections, ideally over a few days. Alternatively, convenient samples of urine must be indexed to the concentration of creatinine to reduce the error associated with variation in urinary output. For animals it is convenient to use a non-absorbable dietary marker such as chromic oxide, which can be measured in both the diet and feces to facilitate calculating the amount of diet corresponding to the fecal sample analyzed for Se.

**Figure 3:**
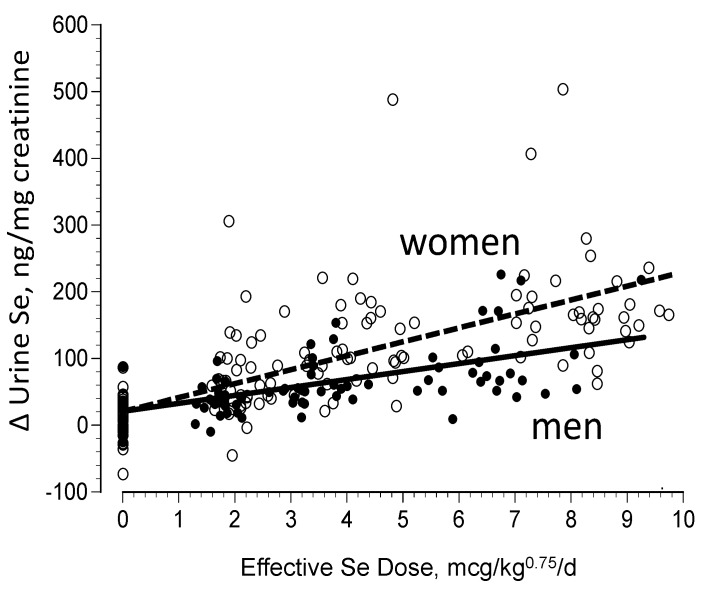
Changes in steady state urinary Se excretion in non-deficient adults after 13 months of supplementation with graded daily doses of SeMet [39].

Excess absorbed Se can be eliminated in the urine by way of metabolism to methylated products including trimethylselenonium ion ([CH_3_]_3_Se^+^) and a selenosugar 1β-methylseleno-*N*-acetyl-d-galactosamine. The dominant chemical form of urinary Se varies among species. In rodents, TMSe^+^ can comprise 90% of urinary Se; in humans, the predominant form is the selenosugar [47,48,49]. In both rodents and humans, urinary Se excretion increases with increasing Se intake [31,40,46,50]. Urinary Se excretion is related to both Se status and the availability of the methyl donor S-adenosylmethionine [51]. Accordingly, urinary Se excretion is negatively associated with plasma homocysteine concentration, but positively associated with plasma concentrations of folate and vitamin B_12_ [50]). Urinary Se excretion appears to differ among males and females, which may be related to sexually dimorphic selenoprotein biosynthesis as has been described in rodents [52]. In a cohort of healthy, non-deficient Americans, we found the dose-dependent urinary Se excretion of women to be 74% greater than that of men Se (Figure 3), despite the fact that both showed similar plasma Se responses to comparable intakes of Se [40]. This indicates that women do not retain SeMet-Se as well as men.

Urinary Se excretion also has a genetic determinant. We found the increase in urinary Se of non-deficient humans with increasing Se intake to be 59% greater for individuals with the *GPX1* 679 T/T genotype compared to those with the *GPX1* 679 C/C genotype [40]; while at baseline, the former individuals had slightly lower plasma Se levels. Because GPX1 accounts for some 60% of tissue Se [53], differences in its turnover are likely to affect Se retention and be manifest as differences in urinary Se excretion.

Hair and toenails have been used to assess long-term Se status in epidemiological studies, offering the advantage of simple, low-cost sample storage. Their analysis calls for careful cleaning, with special concern for hair as to whether subjects may have used selenium sulfide-containing, anti-dandruff shampoos. Selenium has been determined in both types of samples by neutron activation analysis, or by hydride-generation atomic absorption spectrophotometry (HG-AAS) after acid-digestion. With standardized procedures for collecting these samples, both hair and nail Se levels correlate well with blood/plasma Se concentration [54,55]. Their use to assess Se status relies on the implicit assumption that their Se contents reflect some metabolically relevant component of body Se, an assumption that has never been validated. In fact, hair/nail Se is not in dynamic equilibrium with any component of circulating Se; it is actually an excretory form of the element. As such, samples of each represent portions of each that were secreted at some time past, presumably reflecting Se status at that time and making them most useful in studies of populations with stable dietary practices. These contributions are very small in comparison to urinary Se output and can be ignored in estimating Se retention.

Assessment of Se retention is useful in livestock nutrition where it can be used as a measure of the bioavailability of feed forms of Se [56], or as a proxy for the amount of Se in edible tissues. Tissues retain Se largely in protein-bound forms, SeCys being specifically incorporated into selenoproteins and SeMet being nonspecifically incorporated into all proteins (Figure 2). Accordingly, animals fed dietary sources of SeMet will accumulate relatively large amounts of Se in both the specific and non-specific pools; whereas, those fed inorganic Se-compounds, e.g., selenite, will accumulate lesser amounts due to that form only entering the specific (SeCys) pool.

Estimating retained Se is useful in human studies as indicators of the degree to which an individual has met his/her Se requirement and may have Se reserves. This can be inferred from analyses of the two specific selenoproteins (SEPP1, GPX3) as well as the non-specific component of plasma Se. Those selenoproteins show maximal expression at plasma Se concentrations of 50–70 ng/mL [57].

### 3.3. Biomarkers of Tissue Selenium Levels

Selenium can be retained in many organs. Greatest concentrations are found in kidney, liver and pancreas, followed by cardiac and skeletal muscle; this pattern is remarkably similar across species, particularly under non-deficient conditions [56]. Because of its non-specific incorporations into proteins, food sources of SeMet support greater tissue Se accumulation than comparable intakes of Se in inorganic forms. For this reason, animal studies using tissue Se accumulation as the measure of bioavailability typically show Se in foods/feedstuffs to be more highly available than selenite or selenate [56].

Earlier studies employed whole blood for the assessment of Se status (e.g., [55,57]). Those showed good correlations with functional biomarkers in cohorts of relatively low Se status, as well as responsiveness to supplementation with food sources of Se (largely SeMet). Blood samples can be dried and later analyzed by X-ray fluorescence analysis [58]. Nevertheless, whole blood Se measures can be difficult to interpret in as much as they comprise both cellular and non-cellular constituents both of which have specific and non-specific components.

**Figure 4:**
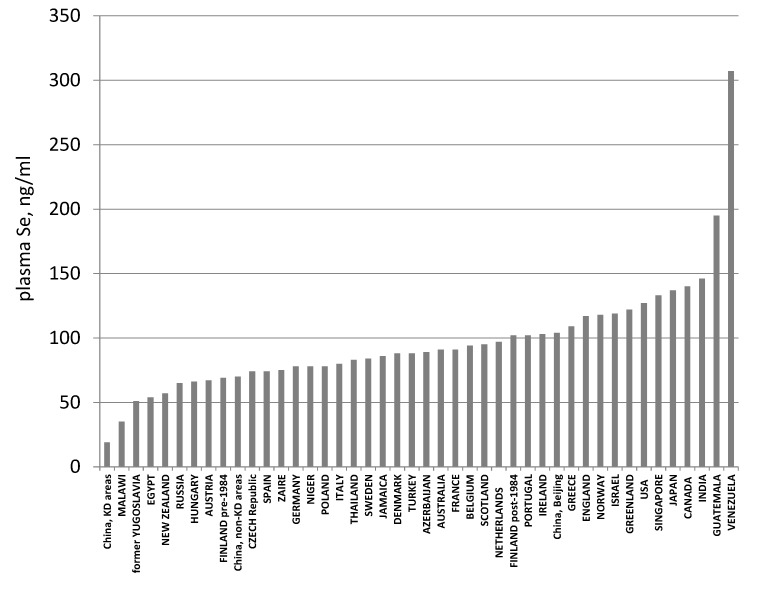
Global variation in Se status as indicated by plasma Se concentration. Note: Because maximal selenoprotein expression is associated with plasma Se concentrations around 80 ng/mL, countries with averages less than *ca.* 100 ng/mL (parts of China, New Zealand, and much of Europe) are likely to have appreciable portions of their populations sub-optimally nourished with respect to Se. After Combs [23].

The most useful tissue for assessing Se status, particularly in humans, is plasma (or serum, which is virtually equivalent with respect to Se-containing components and, therefore, total Se contents; see below). Selenium is stable in plasma as long as microbial growth is prevented; it can be determined with very good sensitivity and precision by automated atomic absorption spectrophotometry reducing interferences by using either HG-AAS or electrothermal (graphite furnace) atomic absorption spectrophotometry (GF-AAS). Studies in many countries have shown the effects of geographic variation in the Se contents of foods (Figure 4). From these it has become clear that: parts of China and Malawi have residents of very low Se status; several other countries have appreciable numbers of subjects with sub-maximal GPX3 expression; certain parts of a few countries have residents of very high Se status; and frank selenosis in humans has been limited to a couple of discrete seleniferous locations.

Plasma Se consists mostly of Se that has been incorporated both specifically and non-specifically into various proteins. Selenocysteine occurs in only two plasma selenoproteins, SEPP1 and GPX3, which contain 10 and 4 SeCys residues, respectively. The synthesis of each, occurring in the liver and kidney respectively, is regulated depending on an ample metabolic supply of selenide for the co-translational conversion of serine to SeCys while the amino acid remains bound to a specific tRNA [59]. Selenoprotein P can be measured by immunoassay [60,61,62]. The GPXs have been assessed by their enzymatic activity, which has typically been measured using a kinetic, spectrophotometric assay coupled to glutathione reductase [63]. The specific Se pool can be calculated from the activity of GPX3 and the mass of SEPP1 (SeCys can be calculated making the following assumptions: GPX3 rate constant of 2.8 × 10^4^ nmol/min/mg, molecular weight 92 kD and stoichiometry of 4 g-atoms Se per mole, as SeCYS [64]; SEPP1 (glycosylated), average molecular weight 60 kD and stoichiometry of 7.5 g-atoms Se per mole as SeCYS [60].) This yields estimates in the nM range.

Plasma also contains a non-specific Se pool comprised of SeMet incorporated into proteins *in lieu* of Met with which it competes for tRNA^Met^ binding. The potential for the unregulated, non-specific incorporation into proteins is a function of the Met contents of those proteins. In plasma, the most quantitatively important of these is albumin, which contains 6 Met residues per mole. Assuming that SeMet can compete for incorporation at each of those positions, that protein would provide a plasma Met space equivalent to nearly 4 mM. The non-specific pool can be estimated from the difference between the specific and total Se pools. By this approach, we found in a non-deficient cohort more than half of plasma Se present in specific components (GPX3 and SEPP1 comprising roughly equal amounts), with almost as much Se present in non-specific components [50]. Further, the increase in plasma Se produced by SeMet-supplementation consisted entirely of expansion of the non-specific component, which reached 73% of plasma Se in subjects consuming some 300 ug Se/day. Still, that amount comprised less than 0.05% of the potential SeMet space, indicating that the latter is unsaturable under practical conditions.

**Figure 5:**
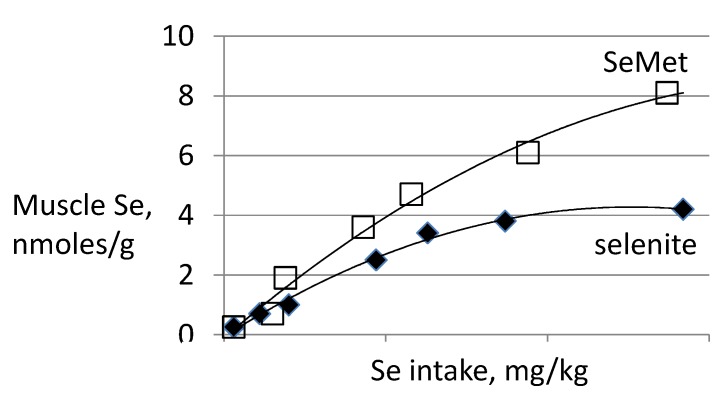

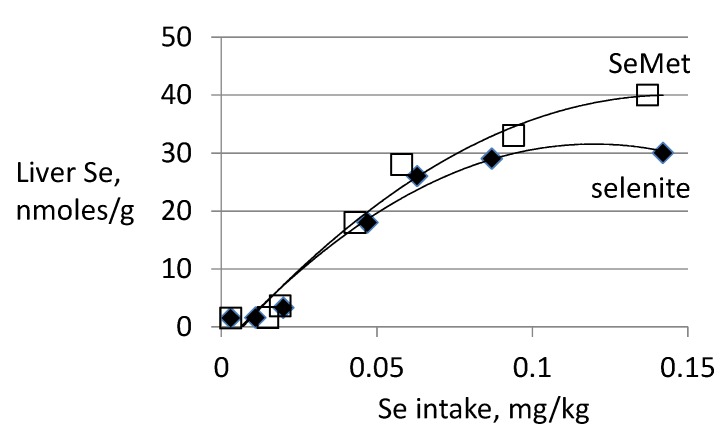
Effect of the form of dietary Se on tissue deposition of Se. After Reeves *et al.* [65].

In contrast to SeCys and selenite, which enter the specific selenoprotein pool by a highly regulated process, the incorporation of SeMet into the nonspecific Se pool is unregulated, its size reflecting the intake of SeMet over a wide range. Accordingly, sources of SeMet are also more effective than inorganic Se in increasing tissue Se levels [33,40,41,42,43,44,57] (e.g., Figure 5).

While Se intake is its primary determinant, plasma Se level can be also affected by other factors affecting any of the Se-containing proteins. Plasma Se concentration has been shown to vary with gender, decline with age, and be reduced in smokers [66] and subjects with protein malnutrition [67] or inflammation [68,69]. We found plasma SEPP1 levels to be somewhat lower in obese individuals (body mass index >30) [50], and others have noted lower SEPP1 concentrations in African Americans compared to White Americans [70].

**Figure 6:**
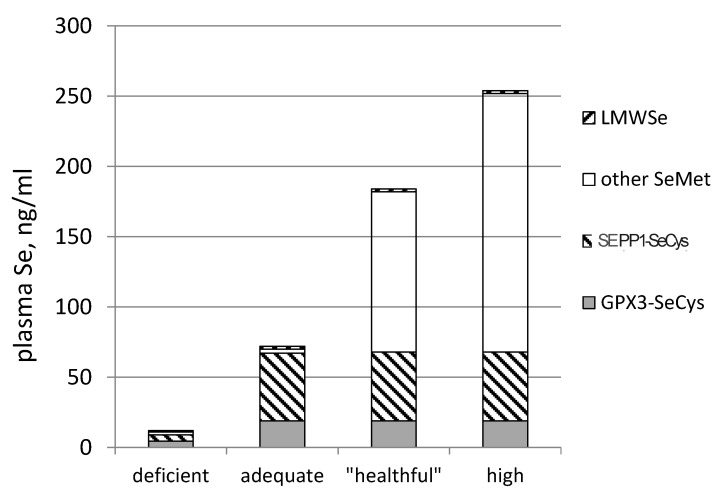
Stylized patterns of specific (solid areas, SeCys) and non-specific (hashed areas, presumed to be mostly SeMet in albumin) major components of plasma Se in subjects varying in Se status by consuming SeMet-containing foods. Not shown: Se occurring as SeMet in GPX3 and SEPP1, as their potential contributions to non-specific plasma Se are very small (<0.1%). Group labels: deficient = less than maximal selenprotein expression; adequate = maximal selenoprotein expression, *i.e.*, meets nutritional requirement; “healthful” = supranutritional, including anti-tumorigenic doses; high = greater than needed for anti-tumorigenesis, but without adverse effects.

The informative value of plasma Se as a biomarker, therefore, depends on Se status. This is depicted in Figure 6. Measurements of GPX3 activity and SEPP1 can identify the risk of nutritional deficiency. Measurements of total Se and, perhaps, the non-specific pool can identify luxus Se status. Thus, it is apparent that the largest contributor to the global variation in plasma Se (Figure 4), particularly among subjects with plasma Se concentrations greater than *ca.* 70 ng/mL, is the non-specific pool.

**Figure 7:**
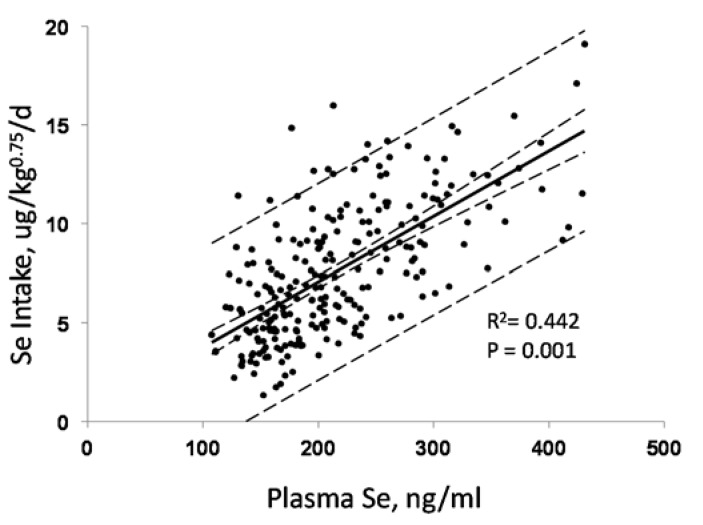
Relationship of plasma Se concentration and estimated Se intake in non-deficient Americans. (Regression: Se_in, μg/kg_0.75_/day_ = 0.44 + 0.03 × Se_plasma, ng/mL_; dotted lines indicate 95% confidence limits) Recalculated from data of Combs *et al.* [50].

For Se-nondeficient individuals consuming Se mostly as SeMet, Se intake can be predicted from the Se concentration of plasma. We estimated Se intake in a cohort of Americans with relatively high baseline plasma Se levels averaging 142 ng/mL [50]. We did this using the amounts of Se provided by administered daily supplements of SeMet plus those coming from the subjects’ diets as estimated from a self-administered food frequency questionnaire and the USDA National Nutrient Database for Standard Reference, Release 27 [71]). Our results showed estimated Se intake (Se_in_, expressed as ug/kg^0.75^/day) to be significantly correlated with measured plasma Se (Se_plasma_, expressed as ng/mL) (Figure 7). From that regression it is possible to predict Se intake.

Three cell types are readily available for use in assessing tissue Se status in humans: erythrocytes, lymphocytes and buccal cells. The Se contents of each respond to SeMet supplementation. Erythrocyte Se requires digestion after which it can be determined by HG/GF-AAS. Because erythrocytes are collected with blood, they seldom offer advantages over plasma as a specimen for assessing Se status based on Se content. Buccal cells offer the possibility of a minimally invasive biomarker of somatic cell Se status. These cells can easily be collected in amounts sufficient for analyzing Se and can be acid-solubilized rather than digested prior to HG/GF-AAS analysis. Nevertheless, few groups to have used buccal cells for this purpose [40,50,72]. In a non-deficient cohort, we found buccal Se concentration to be positively associated with age and nutritional supplement use, and to respond to SeMet supplementation.

Current knowledge of hepatic Se metabolism (Figure 2) would suggest the presence of low molecular weight Se-metabolites in plasma and tissues. Such metabolites would be expected to include: the selenoamino acids, SeMet and SeCys, from the digestion of Se-containing food proteins; the product of selenoamino acid catabolism and obligate precursor for selenoprotein synthesis, selenide; the methylated products of Se metabolism, TMSe^+^ and selenosugars. In plasma, such metabolites would appear to comprise only a small portion (<2%) of plasma Se. Until recently they have eluded identification. Gammelgaard’s group was able to speciate low molecular weight metabolites in biological samples using liquid chromatography/inductively coupled plasma mass spectrometry (LC-ICP-MS). They identified TMSe^+^, methyl-SeCys, SeMet and the selenosugar Se-methyl-*N*-acetylselenohexamine in tissues of the Se-fed rat [51]. They found no quantifiable Se-metabolites in plasma, but significant amounts of TMSe^+^, the selenosugar, methyl-SeCys and SeMet in liver and kidney, and low concentrations of TMSe^+^, SeMet and selenosugar in erythrocytes.

### 3.4. Biomarkers of Se Function

The nutritional role of Se appears to be discharged by a group of selenoproteins, which are encoded by only 25 genes [73]. Most, if not all, selenoproteins of which are involved in the regulation of redox signaling [74]. The best understood and most abundant members of the human selenoproteome include:

- Glutathione Peroxidases (GPXs), which reduce hydroperoxides using reducing equivalents from reduced glutathione [75];
- Thioredoxin Reductases (TXNRDs), NADPH-dependent flavoenzymes that function in intracellular redox regulation by reducing thioredoxin [76];
- Iodothyronine Deiodinases (DIOs), which remove iodine from the thyroid hormones (T_4_, T_3_) in the metabolism of the active hormone, T_3_ [77]; and
- SEPP1, produced and excreted by the liver, functioning as the primary transporter of Se to peripheral tissues, and comprising 40%–60% of total plasma Se [46].

In principle, measurement of any of these selenoproteins would yield information immediately relevant to the physiological function of Se as an essential nutrient. Those that have been most widely used as biomarkers in animal studies are the intracellular and extracellular GPXs, GPX1 and GPX3, respectively. These, the TXNRDs, and the DIOs show maximal expression in most species with dietary Se concentrations of 0.1–0.2 mg/kg [78,79,80]. Only a single selenoprotein, SEPW1, is known not to be maximally expressed under such conditions, but to be upregulated by doses of Se in the anti-tumorigenic range [81].

The list of biomarkers of Se function available for human studies is much shorter, being limited to those occurring in accessible tissues: GPX3, which comprises 10%–25% of plasma Se (lower levels being associated with greater plasma Se concentrations); SEPP1, which comprises 20%–70% of plasma Se (lower percentages being associated with greater plasma Se concentrations); and GPX1, which can be assayed in erythrocytes, lymphocytes, buccal cells and tissue biopsy specimens.

A large amount of early work involved erythrocyte GPX1, the first selenoezyme discovered [82]. Its activity is measured spectrophotometrically by a glutathione reductase coupled assay [63]. Because heme iron can cause interfering glutathione oxidation in that assay, it is necessary to treat erythrocyte samples with Drabkin’s reagent. The selenoprotein is a homotetramer; each 23 kD subunit contains a SeCys residue at its active center. More recent studies have relied on the extracellular isoform, GPX3, which is of simlar size and stoichiometry but, unlike GPX1, is a glycoprotein produced mostly by the kidney. Selenoprotein P is present in plasma as multiple variants with an average molecular weight of 60 kD [64,83,84,85]. Although its genetic coding would suggest as many as 10 SeCys residues per mole [86], studies in the rat have shown only 7–8 residues per mole [87].

The dependency of the expression of these selenoproteins on Se intake has been demonstrated in humans for GPX3 [88,89,90,91] and SEPP1 [90,92] in studies conducted in parts of China with endemic Se deficiency. In both cases, pooled analyses of subjects from different communities with differing plasma Se concentrations showed GPX3 activity and plasma SEPP1 concentration each to be directly related to plasma Se concentration over ranges of about 8–80 ng Se/mL. This work has provided the basis for setting dietary Se requirements, which have been based on either two-thirds or maximal GPX3 expression, yielding dietary recommendations for adults from 26 to 70 μg Se/day [24].

**Figure 8:**
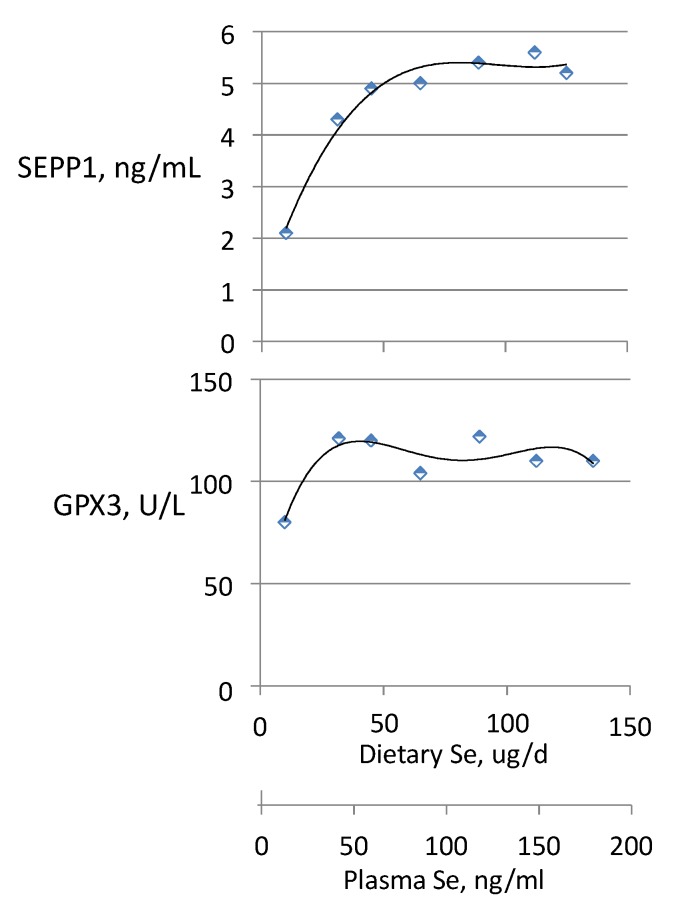
Relationships of GPX3 activity and SEPP1 concentration to dietary Se intake and plasma Se concentration in residents of a low-Se part of China. Subjects had an estimated average intake of 10 μg/day from dietary sources and had been supplemented with graded amounts of SeMet for 40 weeks. Estimated from the data of Xia *et al.* [91].

Maximal GPX3 expression may occur at a lower level of Se status than maximal SEPP1 expression. This was suggested by the data of Xia *et al.* [32], which showed that Se-deficient subjects supplemented with SeMet reached maximal GPX3 activities when their plasma Se concentrations were around 66 ng/mL, but that their plasma SEPP1 concentrations did not appear to be maximally expressed until their plasma Se concentrations were at least a third higher (Figure 8). However, the SeMet intervention period in that study was relatively short (20 weeks), and it is likely that SEPP1 may have taken longer to reach new steady-state levels. When the same group used a longer intervention period (40 weeks), the threshold of maximal expression of GPX3 was slightly lower than that of SEPP1. Both thresholds were in the range of Se intakes of 35–45 mcg/day, corresponding to plasma Se concentrations of 50–70 ng/mL [90] (Figure 8). These thresholds are typically exceeded in western populations (Figure 4); Nève [92] noted that subjects with plasma Se concentrations >70 ng/mL do not show GPX3 responses to Se-supplementation. Accordingly, negative GPX3 and SEPP1 responses to Se supplementation are typical in trials conducted in cohorts with greater Se intakes such as in the US [35,39] and UK [38].

It is possible that obesity affects SEPP1 expression. In a cohort of non-deficient subjects we found plasma SEPP1 expression to be slightly depressed among subjects with body mass index (BMI) great than 30. This is consistent with that of Méplan *et al.* [93] who found relatively low-Se (average plasma Se 90 ng/mL) subjects with BMI > 30 to have lower baseline plasma Se concentrations than non-obese subjects; obese females also showed lower GPX3 activities. These findings may reflect influences of pro-inflammatory cytokines, which have been found to inhibit SEPP1 expression [94], or dysregulation of gluconeogenesis, as SEPP1 appears to be regulated as a gluconeogenic enzyme [95,96].

Non-specific parameters have been used to assess metabolic impacts of Se deficiency. The ratio of thyroxine (T4) to active thyroid hormone triiodothyronine (T3) in the plasma, normally about 20:1, is elevated by Se-deficiency [97,98]. This results from limited expression of the DIOs; hence, Se-deprivation can contribute to iodine deficiency diseases. While useful as an indicator of thyroid hormone status, the T3:T4 ratio lacks specificity for Se status, as not only can the conversion of T4 to T3 be impaired by Se deprivation, but also the synthesis of T4 can be impaired by deprivation of Se and/or iodine.

Genetic variability and gender contribute to variance in biomarkers of Se function. We found individuals with *GPX1* 679T/T genotype (which has been associated with increased risk to certain cancers [99,100]) to have lower plasma Se levels than those with *GPX1* 679C/C alleles; individuals with the *SEPP1* 24731 A/A genotype to have 27% greater levels of plasma SEPP1 than those with the *SEPP1* 24731 G/A or G/G genotypes; and individuals with the *SEP15* 811 T/C genotype to have greater buccal cell Se levels than those with the *SEP15* 811 C/C genotype [50]. Hurst *et al.* [38] found two variants of SEPP1 to affect circulating concentrations of selenoproteins. One variant at position 234 of the protein may affect SEPP1 turnover; the other variant at r25191 in the 3’-untranslated region may affect Se incorporation into SEPP1. They found women with the *SEPP1* 234GA genotype showed lower SEPP1 levels than men of the same genotype; while women with the *SEPP1* 234GG genotype showed lower SEPP1 levels than men of that genotype. Subjects with the *SEPP1* 234AA genotype showed lower plasma TR1 concentrations compared to those of the other genotypes. Variants in r25191 did not affect SEPP1 levels; however, individuals with the r25191GG genotype showed greater plasma TR1 concentrations than women of that genotype, while males of the r25191GA genotype were the only subjects to show TR1 responses to Se supplementation.

It is possible that dietary Se may also affect the microbiome of the hindgut, which is involved in immunologic signaling, energy abstraction from non-digested carbohydrates, and carcinogenic risk. Selenium reaches the hindgut in the form of non-digested Se from the diet and endogenous Se from enterohepatic circulation and sloughed mucosal cells. Dietary Se has been shown to promote taxonomic diversity of the microbiome of the rat [101], which has been found capable of producing methylated Se-metabolites independent of the host [102]. That the pattern of low molecular weight Se-metabolites in the hindgut lumen corresponds with that those of feces [103], would appear to making fecal sample useful as an indicator of the Se in the colon. Because Se is required by several methanogenic *Archaea* and gram^+^ bacteria that express selenoproteins [104,105,106,107,108], it is possible that these species may be growth-limited by Se supply. The few relevant studies conducted to date provide some support for this hypothesis [109,110].

### 3.5. Biomarkers Relevant to Antitumorigenesis

Studies with animal models have demonstrated anti-tumorigenic effects of Se involving several aspects of cell proliferation, migration and apoptosis [7]. These effects are manifest with dietary Se levels of at least 1.5 mg/kg, while most selenoproteins are maximally expressed at far lower dietary levels (0.1–0.2 mg/kg). For that reason, we proposed that the anti-tumorigenic effects of supranutritional doses of Se must involve functions of Se-metabolites and not of selenoproteins [111]. It must be recognized, however, that current knowledge of the selenoprotein responses to Se exposure are limited to a few tissues, particularly, liver, kidney, skeletal muscle. It is possible that maximal expression of selenoproteins in particular cell types, e.g., cytotoxic killer cells, may require greater Se exposures. That certain selenoproteins may play roles in Se-antitumorigenesis is suggested by the finding of Hawkes *et al.* [81] that the mRNA for SEPW1 is up-regulated by supranutritional Se treatment, and that silencing SEPW1 is associated with reduced stability of the tumor suppressor protein p53, suggesting that SEPW1 may serve in its stabilization [112].

Studies by Ip, Ganther and colleagues [21,113] have indicated methylselenol (CH_3_SeH) as an active principle in Se-antitumorigenesis. Methylselenol is produced by the SAM-dependent methylation of hydrogen selenide in the rate-limiting step in the ultimate production of TMSe^+^ [114] (Figure 2). Methylselenol has been found to modulate the expression of key genes in the p53 pathway [115] and the lymphocyte cell surface ligands that trigger immune activation [116] and to reduce plasma concentrations of proteases and angiogenic factors required for tumor spreading [8,9,117]. The involvement of CH_3_SeH and perhaps other methylated Se-metabolites in Se-antitumorigenesis suggests possible underlying mechanisms which may become metabolically significant at supranutritional Se intakes: redox cycling of H_2_Se and/or CH_3_SeH to generate reactive oxygen species (ROS); modifications of protein-thiols by H_2_Se and/or CH_3_SeH to affect protein function/turnover; Met mimicry by SeMet in proteins to sensitize critical regulatory proteins to ROS [118].

Evidence from human trials is consistent with the view of anti-tumorigenic Se-metabolites, showing reduction in cancer risk among subjects of nutritionally adequate (*i.e.*, maximal GPX3 expression) but not high Se status. For example, the Nutritional Prevention of Cancer Trial [17,18] found supplemental Se to reduce cancer risk in non-deficient Americans only if their baseline plasma Se levels were less than *ca.* 120 ng/mL. This is consistent with the results of the larger Selenium and Vitamin E Cancer Prevention Trial (SELECT) [16], which found no risk reduction in a cohort with baseline plasma Se concentrations exceeding that level.

Although methylated Se-metabolites should be candidate biomarkers relevant to Se-antitumorigenesis, none have been reported in biological specimens. Methods capable of detecting very low concentrations of low molecular weight Se-metabolites have been developed only recently [47,51]. At the moment, therefore, the most useful biomarker relevant to Se-antitumorigenesis is plasma Se, with a target level of *ca.* 120 ng/mL above which current evidence indicates no further risk reduction.

### 3.6. Biomarkers Relevant to Adverse Effects

#### 3.6.1. Selenosis

The potential for very high Se status to produce adverse physiological effects has been established from animal studies and accidental exposures of humans. These reports have produced an array of clinical indicators but few biomarkers with predictive value. For this reason, the default choice has been to use as risk indicators the highest Se tissue levels observed with no adverse effects. Most studies show no adverse effects in human subjects with plasma Se levels <1000 ng/mL.

Acute selenosis has occurred in humans due to accidental ingestion of gram quantities of Se in the form of such high Se-solutions as gun bluing, sheep drench and anti-dandruff shampoo [119]. Each case involved rapid development of severe gastrointestinal and neurological symptoms followed by acute respiratory failure, myocardial infarction and renal failure. Some cases showed garlic odor of the breath due to the excretion of volatile Se-metabolites across the lung [120,121,122]. Garlic breath was among the earliest signs reported for selenotic animals [123,124] and is due mostly to the presence of dimethylselenide ((CH_3_)_2_Se) excreted across the lung. In rodents, injected selenite causes a dose-dependent increases in the excretion of (CH_3_)_2_Se [125], even at sub-toxic doses at which it can represent nearly 30% of the dose [126,127,128,129,130]. Methylselenol can be measured in breath after a loading dose of ^77^SeMet by cryotrapping followed by gas chromatography-inductively coupled plasma mass spectrometry (GC-ICP-MS) [131,132]. However, such methods have not been used in cases of selenosis and diagnostic criteria have not been established.

Chronic selenosis was described in a small, mountainous part of eastern China where residents had consumed foods containing very high levels of Se (several hundred mg/kg) due to the use of the ash from local high-Se coal ash to amend agricultural soils [133,134]. Affected individuals showed hair loss, pathological nails and some elevated skin sensitivity; in all cases, symptoms resolved when Se intake was reduced. No individual with Se intakes less 1500 μg/day showed adverse effects. The US Food and Drug Administration used these cases to set a “no observed adverse effect level” (NOAEL) for Se at a whole blood Se concentration of 1000 ng/mL, which they calculated to correspond to a dietary intake of 853 μg/day in an adult male [135]. It is likely that plasma Se concentrations of that magnitude are comprised of very large non-specific components and, perhaps, enlarged low molecular weight components, although there is no relevant empirical evidence. The Food and Nutrition Board [119] set a tolerable upper Se intake level (UL) for adults at 400 μg/day, *i.e.*, about half the NOAEL; other national bodies adopted similar recommendations, *i.e.*, 350–450 μg/day for adults [24].

#### 3.6.2. Type 2 Diabetes Risk

Some studies have associated plasma Se concentrations exceeding *ca.* 140 ng/mL with increased risk of type 2 diabetes (T2D) [136,137]. Those findings were based on self-reports of T2D and/or medication use consistent with T2D, unconfirmed with measures of circulating glucose, insulin or glycated hemoglobin (HbA1c). They have not been confirmed in other randomized Se-intervention trials [16,40,138,139,140], including one in which subjects showed plasma Se concentrations exceeding 200 ng/mL [40].

Some studies with animal models have yielded supporting results. A comparison of supranutritional (3 mg Se/kg diet) and nutritional (0.3 mg Se/kg diet) intakes of Se in gestating rats and their offspring found the high-level Se exposure to induce insulin resistance and glucose intolerance late in gestation and in offspring after several weeks postpartum [141]. A similar experiment with pigs found fed a diet containing 3 mg Se/kg to support greater circulating insulin levels, with normal glucose levels, compared to those fed the nutritional level [142].

That selenproteins may be involved in glycemic control has been suggested by findings of perturbations in glucose metabolism due to Se-deprivation [143,144], limited synthesis of multiple selenoproteins [145], and GPX1 overexpression [146] in animal models. Such effects might be expected, as the GPX1 substrate, H_2_O_2_, is known to participate in insulin signaling by oxidizing phosphatase PTEN. Similarly, the synthesis of SEPP1 is known to be suppressed by insulin and upregulated under conditions of hyperglycemia [147]. Still, it seems unlikely that such findings are relevant to the prospect of high-Se exposures increasing T2D risk, as selenoproteins are maximally expressed at levels of Se intake lower those purportedly associated with T2D risk. Therefore, while it would appear prudent to monitor fasting glucose, insulin and HbA1c in intervention trials with supranutritonal levels of Se, the most useful Se-biomarker for this putative risk remains plasma Se concentration with a practical upper limit that would appear to be no lower than 300 ng/mL.

## 4. Summary

The multiple biological activities of Se call for biomarkers that can provide information about Se status relative to nutritional (functional) needs, anti-tumorigenic potential, and risk of adverse effects (Table 1). Accordingly, Se-biomarkers of two types:

1. Se-biomarkers (e.g., GPX3, GPX1 and SEPP1) that provide information about function directly. They are useful under conditions of Se intake falling within the range of regulated selenoprotein expression, which for humans is less than about 55 μg/day (in parts of China, New Zealand and Europe), and for animals is less than about 0.1 mg/kg diet. Their values are in identifying nutritional Se deficiency and tracking responses of deficient individuals to Se-treatment.
2. Se-biomarkers that provide information indirectly through inferences based on Se levels of foods, tissues, urine or feces. For example, SeMet in plasma and tissues can indicate amount of Se potentially available for functional use, the likelihood of Se-deficiency or the likelihood adverse effects of Se, but it provides no direct evidence of either of these states. The value of these biomarkers is in providing information about Se status over a wide range of Se intake, particularly from food forms, over which tissue retention of SeMet is unregulated.

Additional biomarkers of the first type are needed to improve the sensitivity and specificity of assessing Se status in non-deficient individuals for whom cancer risk reduction and risks of adverse effects are the primary health considerations. This agenda must include investigation and method development. Studies are needed to learn how/whether supranutritional Se status may affect cancer immunosurveillance. Might maximal selenoprotein expression by immune cells (e.g., B cells, T cells, natural killer cells, natural killer T cells, macrophages, dendritic cells) require supranutritional exposures to Se? If so, then assessment of selenoproteins in such blood cell types could be a valuable biomarker relevant to Se-anti-tumorigenesis.

**Table 1:** Summary of Selenium Biomarkers.

Component of Se Status	Specimen	Se Biomarker	Informative Value	Limitations
Se Intake	foods	Se form and amount; amount of food consumed	Approximates total Se consumed.	Use of food nutrient data bases do not address regional variation or digestibility.
Tissue Se	Humans: whole blood, plasma/serum; erythrocytes, buccal cells, lymphocytes, nails, hair; Animals: *also* liver, muscle, other tissues	total Se	Indicates portion of ingested Se absorbed and retained. Most useful in animal studies in which larger body Se pools (liver, muscle) can be sampled.	nail, hair: Samples represent past Se status; collection must be standardized; samples subject to contamination
		non-specific protein-SeMet	Indicates portion of retained Se that may become available for functional purposes over medium-long term.	Must be imputed from Se and selenoprotein contents of tissues. Can be approximated by albumin-Se (few supporting data).
Se Function	Humans: whole blood, plasma/serum; erythrocytes, buccal cells, lymphocytes; Animals: *also* liver, muscle, other tissues	*nutritional:* selenoproteins	Indicates Se function, portion of retained Se in functional forms. In humans, GPX3, GPX1 and SEPP1 are most useful—can be measured in plasma, and blood/buccal cells.	Assays established for GPX’s, SEPP1, TXNRDs and DIOs.
		*antitumor:* Se-metabolites, e.g., CH_3_SeH	(Would indicate amount of Se antitumorigenically active.) *	CH_3_SeH has not been detected in tissues.
		*adverse effects:* Se-metabolites, e.g., (CH_3_)_x_Se	(Would indicate Se function and portion of retained Se in functional forms.) *	Methylated Se-metabolites have not been detected in tissues.
Se Excretion	urine	total Se	Indicates major portion of absorbed Se not retained.	
		Se-sugar	Major form of excreted Se in humans.	Minor component of excreted Se in animals (rodents).
		(CH_3_)_3_Se^+^	Major form of excreted Se in animals (rodents).	Minor component of excreted Se in humans.
	feces	total Se	Indicates amount of Se available to the hindgut microbiome.	Does not inform re functional effects on the microbiome.
	breath	(CH_3_)_2_Se	(Would indicate exposure to potentially intoxicating levels of Se.) *	Diagnostic criteria of selenosis not established. Confounders: low methyl status, folate, vit. B_12_; methylmercury exposure.

* Methodology not currently available.

Methods are also needed to determine low molecular weight Se-metabolites:

- *selenoamino acids*—The capability to determine SeCys and SeMet in the circulation would facilitate studies of factors affecting the absorption and utilization of Se in foods.
- *methylated Se-metabolites*—The capability to determine (CH_3_)_2_Se and derivatives in the circulation and/or tissues would facilitate the assessment of their roles as putative mediators of Se anti-turmorigenesis as well as of adverse effects of high-level Se exposure.
- *breath (CH_3_)_2_Se*—Methods are already available to determine *(CH_3_)_2_Se* in breath; however, they have yet to be employed to produce dose-response data over a range of human Se exposures that include selenosis. Such data are needed to facilitate use of this parameter as a biomarker of adverse effects risk. Research is also needed to assess potential confounding effects of factors that can enhance (e.g., methylmercury [128]) or impair (e.g., limited methylation capacity [131]) (CH_3_)_2_Se production.

Recent analytical advances using tandem liquid chromatography-mass spectrometry (GC-MS) [47,51,132,133] suggest possibilities in this area.
